# Armed Therapeutic Viruses – A Disruptive Therapy on the Horizon of Cancer Immunotherapy

**DOI:** 10.3389/fimmu.2014.00074

**Published:** 2014-02-24

**Authors:** Maxine Bauzon, Terry Hermiston

**Affiliations:** ^1^Bayer HealthCare, US Innovation Center, Biologics Research, San Francisco, CA, USA

**Keywords:** oncolytic virus, cancer immunotherapy, immune-checkpoint inhibitors, CTLA-4, PD1, PDL1, PDL2, blockade of checkpoint inhibitors

## Abstract

For the past 150 years cancer immunotherapy has been largely a theoretical hope that recently has begun to show potential as a highly impactful treatment for various cancers. In particular, the identification and targeting of immune checkpoints have given rise to exciting data suggesting that this strategy has the potential to activate sustained antitumor immunity. It is likely that this approach, like other anti-cancer strategies before it, will benefit from co-administration with an additional therapeutic and that it is this combination therapy that may generate the greatest clinical outcome for the patient. In this regard, oncolytic viruses are a therapeutic moiety that is well suited to deliver and augment these immune-modulating therapies in a highly targeted and economically advantageous way over current treatment. In this review, we discuss the blockade of immune checkpoints, how oncolytic viruses complement and extend these therapies, and speculate on how this combination will uniquely impact the future of cancer immunotherapy.

## Introduction

Tumors are difficult to treat and in many instances lethal. The treatment challenge is not surprising as they are genetically unstable and complex biological systems with an ability to adapt to and thrive in often harsh and changing environments. Furthermore, this plasticity increases the probability that subpopulations will acquire resistance to any one therapy. Thus one could argue that a disease with such a complex etiology must be met with an equally complex therapeutic approach. Appropriately, oncologists have for some time combined chemotherapy, radiation and surgery and complemented these strategies with more targeted approaches such as tumor selective antibodies and/or small molecule kinase inhibitors (1). More recently, two alternative therapeutic approaches, cancer immunotherapy and oncolytic viruses, have begun to show promise that should further complement the oncologist’s repertoire of anti-cancer agents.

The area of cancer immunotherapy has had a long and complex history (2, 3). The idea that a patient’s own immune system could remove a tumor in much the same way it so efficiently removes invading microbes has been around for more than a century. Through the years, however, this concept of immunosurveillance has fallen in and out of favor perhaps appropriately given the complex and dynamic role, it is now believed to play in cancer, acting anywhere from anti to pro-tumorigenic (4–6). Research is beginning to elucidate the mechanisms by which tumors evade the immune system and in some instances how tumors use it to their advantage. From this research several promising immune-checkpoint inhibitor targets that are now translating into exciting clinical trial results have emerged (7–9).

Like cancer immunotherapy, the concept of oncolytic viruses is not new dating back to at least the beginning of the twentieth century when it was observed that on occasion tumor regression would follow a viral infection (10, 11). Although over 100 years have passed since these initial observations, the idea of using a replicating virus to selectively infect and kill tumor cells remains understandably appealing. Theoretically, either naturally or through genetic engineering, such an agent would spare normal neighboring cells while killing cancer cells by viral lysis. Furthermore, the progeny released from the lysed cancer cells would result in a self-perpetuating and amplifying therapy. Adding to their appeal is the ability of such agents to deliver exogenous genetic material whose product or products could augment the oncolytic viral treatment (12–14). Despite their theoretical promise, the reality is that oncolytic viruses have had limited clinical success as monotherapies perhaps due to an imbalanced focus on safety over potency. Recently however, there are several late-stage clinical trials showing promise which may eventually lead to clinical acceptance (15, 16).

Here, we suggest merging immune-checkpoint blockers with oncolytic viruses. We will discuss not only how these approaches could complement one another biologically for increased therapeutic benefit, but also how they may represent a unique opportunity to employ alternative biological formats not normally utilized commercially (e.g., Fabs, scFv) to increase both the safety and therapeutic profile of these agents. Finally we will touch upon how, together, these attributes might translate into a more economically appealing and clinically active therapy resulting in a truly new and disruptive treatment for malignancies.

## Cancer Immunotherapy-Blockade of Immune Checkpoints

Immunotherapy works to direct the extensive repertoire of the host immune system to fight cancer. This approach strives to stimulate tumor suppression by (a) boosting the patient’s immune system, (b) decreasing the cancer-induced immunosuppression, and/or (c) increasing the immunogenicity of the tumor itself. If the immune system’s ability to rapidly respond to and clear invading microorganisms could be extended to malignant cells then a powerful therapeutic may be realized. Such an approach may hold greater potential than current treatment approaches as it may prove to be more potent, benefit many more cancer types, offer long-lasting protection against the disease, and come with fewer off-target effects. Advances in cellular and molecular immunology have provided enormous insight into the inter-play between tumors and immune cells and from this research have come strategies by which the immune system might be harnessed to fight cancer (7).

The blockade of immune checkpoints is a more recent approach taken to decrease cancer-induced immunosuppression. Immune checkpoints refer to a number of inhibitory pathways that play crucial roles in maintaining self-tolerance and immune homeostasis. Their function is to down-regulate T-cell signaling in order to prevent uncontrolled T-cell proliferation thereby protecting tissues from auto-immune damage while maintaining tolerance to self-antigens. It is becoming increasingly clear that tumors commandeer certain immune-checkpoint pathways particularly against T cells that are specific for tumor antigens. Preclinical and clinical data have demonstrated that this is a major mechanism utilized by the tumor to evade the immune system. If this could be reversed, the resulting amplification of T cells and their activity would be highly beneficial to the patient given the central role T cells play in cell-mediated immunity. The immune checkpoints are controlled by ligand–receptor interactions, which can be readily blocked by antibodies or disrupted by recombinant forms of ligands or receptors making them appealing therapeutic targets. For a list of immune-checkpoint targeting antibodies that are currently in clinical trial see Table 1.

**Table 1:** **The most advanced clinically evaluated immune-checkpoint blocking antibodies**.

Target	Antibody in development	Current clinical status	Reference
CTLA-4	Ipilimumab (MDX-010)	Approved for melanoma 2012. Multiple cancers (phase I, II, III)	(17–19)
	Tremelimumab (CP-675,206)	Multiple cancers (phase I, II)	(20–22)
PD1	Nivolumab (BMS-936558 or MDX1106)	Multiple cancers (phase I, II) Melanoma (recruiting phase III)	(23–25)
	CT-011	Multiple cancers (phase I, II)	(26, 27)
	MK-3475	Multiple cancers (phase I, II, III)	(28, 29)
PDL1	MDX-1105 (BMS-936559)	Multiple cancers (phase I)	(29)
	MPDL3280A	Multiple cancers (phase I, II)	(30)
	MSB0010718C	Multiple cancers (phase I)	
PDL2	rHIgM12B7	Melanoma (phase I)	
B7-H3	MGA271	Multiple cancers (phase I)	(31)
		Melanoma (phase I)	
LAG3	BMS-986016	Multiple cancers (phase I)	

*Above trial information from ClinicalTrials.gov*.

The inhibitory receptor, Cytotoxic T-lymphocyte-associated antigen 4 (CTLA-4), was the first checkpoint receptor to be extensively and successfully pursued as an anti-cancer target (32). The primary function of CTLA-4 is to regulate the magnitude of T-cell activation. It is expressed solely on T cells where it offsets the actions of CD28, a T-cell co-stimulatory receptor. Because CTLA-4 has a higher affinity for the CD28 ligands B7.1 and B7.2 it, in effect, out-competes CD28 for ligand binding resulting in an attenuated T-cell response (33–37). The lethal systemic immune hyperactivation phenotype of *Ctla4*-knockout mice clearly shows the importance of CTLA-4 and the need to keep T cells in check (38, 39). In 2011, an antibody against CTLA-4 (ipilimumab) was given FDA approval for the treatment of metastatic melanoma (20, 40–42). In a pivotal phase III randomized three-arm clinical trial, melanoma patients were treated with a glycoprotein 100 (gp100) peptide vaccine alone, ipilimumab alone, or the gp100 peptide and ipilimumab. Both ipilimumab groups demonstrated an increased survival of 3.5 months compared with the group receiving the gp100 peptide alone. Moreover, long-term survival was greatly increased with 18% of patients receiving ipilimumab surviving for greater than 2 years as compared with only 5% for the gp100 peptide alone cohort (17). Although ipilimumab treatment was relatively brief, spanning only 3 months, the finding of long-term progression-free survival supports the idea that immune-based therapies may actually result in a reprogramed immune system which can confer long-term antitumor immunity. Clinical trials are on-going evaluating the use of anti CTLA-4 antibodies in other cancer indications including lung, colorectal, renal, and ovarian (43).

The immune-checkpoint receptor, programed cell death 1 (PD1) and its ligands PDL1 and PLD2, are also emerging as promising targets. PD1 like CTLA-4 plays a role in regulating and maintaining the balance between T-cell activation and tolerance (44, 45). However, unlike CTLA-4, PD1 is more broadly expressed and can be found on other activated non-T-lymphocyte subsets including B cells and natural killer (NK) cells. Additionally while CTLA-4 primarily regulates T-cell activation, PD1 principally controls T-cell activity (46). The ligands PDL1 and PDL2 are commonly upregulated on the surface of many different human tumors with PDL1 being the predominant PD1 ligand on solid tumors. High expression levels of PDL1 have been shown on melanoma, lung, ovarian, and other human cancers (47, 48). PDL1 is also expressed on myeloid cells in the tumor microenvironment. *Pdl, Pdl1*, and *Pdl2*-knockout mice demonstrate a milder auto-immune phenotype than *Ctla4*-knockout mice (49–52). Preclinical studies have shown that blocking PD1 or its ligand PDL1 enhances immunity *in vitro* and mediates antitumor activity in preclinical models (53–55). Although the development of PD1 targeting antibodies is not as mature as that of CTLA-4 antibodies, preliminary clinical results look encouraging. In phase I trials of an anti-PD1 antibody (nivolumab), objective responses (complete or partial responses) were observed in those with non-small-cell lung cancer, melanoma, or renal-cell cancer with cumulative response rates ranging from 18 to 28%. Responses were durable with 20 of 31 responses lasting 1 year or more (56). In a separate phase I trial of patients with various advanced cancers, an anti-PDL1 antibody (MDX-1105) also induced durable tumor regression (objective response rate, 6–17%) and prolonged stabilization of disease (12–41% at 24 week) (57).

Beyond CTLA-4 and PD1, molecular immunology has begun to reveal additional receptors and ligands that serve an inhibitory immune function. These include B and T-lymphocyte attenuator (BTLA), T-cell membrane protein 3 (TIM3), Lymphocyte activation gene 3 (LAG3), adenosine A2a receptor (A2aR), and the B7 family of inhibitory ligands (58–66). Each has been associated with the inhibition of lymphocyte activity in preclinical models and consequently antibodies against a number of these targets are being actively pursued (58–66). Additionally, because multiple inhibitory ligands and receptors contribute to the tumor’s evasion of the immune system and appear to be non-redundant, there remains the possibility of further enhancing antitumor immunity by blocking multiple immune checkpoints. Currently several preclinical and clinical studies are on-going testing the effects of blocking a combination of immune checkpoints (Table 2) (67–73). In fact, a recently published phase I study in patients with melanoma that combined anti-CTLA-4 (ipilimumab) and anti-PD1(nivolumab) mAbs resulted in a rapid and deep tumor regression in a substantial proportion of patients (53% of patients had an objective response, all with tumor reduction of 80% or more) (74). These objective response rates exceeded the previously reported results with either mAb alone (17, 56).

**Table 2:** **The current clinical development of combined immune-checkpoint targeting agents**.

Stage of clinical development	Targets	Antibodies in development	Target disease
Phase III	CTLA-4/PD-1	Ipilimumab + Nivolumab	Metastatic melanoma
Phase II	CTLA-4/PD-1	Ipilimumab + Nivolumab	Metastatic melanoma
Phase I	CTLA-4/PD-1	Ipilimumab + Nivolumab	Metastatic renal-cell carcinoma
	CTLA-4/PD-1	Ipilimumab + Nivolumab	Malignant melanoma
	CTLA-4/PD-1	Ipilimumab + Nivolumab	Non-small-cell lung cancer
	LAG3/PD-1	BMS-986016 + Nivolumab	Multiple cancers

*Above trial information from ClinicalTrials.gov*.

## Oncolytic Viruses as (Immuno)Therapies

Oncolytic viruses can be RNA or DNA based and derived from human (e.g., herpes simplex virus, adenovirus, measles virus) or animal [e.g., vesicular stomatitis virus (VSV), Newcastle disease virus, myxoma virus] viruses. By definition they selectively replicate in, and kill cancer cells. This selectivity can be a natural property of the virus or an engineered trait (75–81). Oncolytic viruses can also be genetically armed to improve or generate more tumor selective cell killing. For example, cell death can be induced by delivering tumor-suppressors (e.g., p53, p16), pro-apoptotic proteins (e.g., TRAIL, IL-24), or small hairpin RNA targeting cell survival or proliferation factors (e.g., hTERT, survivin) (82–87). Arming can also sensitize the tumor to chemo or radiotherapy (Prodrug enzymes, NIS) (88–90).

Although direct oncolysis was envisioned as the primary desired outcome of this therapeutic approach, research and clinical data is supporting the assertion that these productive tumor-specific infections can elicit additional antitumor effects. For example there is evidence that oncolytic viral therapy can induce tumor vasculature shutdown resulting in tumor necrosis (91, 92). Data also suggests that because oncolytic viruses result in highly pro-inflammatory and immunogenic events (tumor cell death and the release of tumor-specific antigens) (93–95) they can elicit a tumor-specific immune response (96). Additionally, viruses encode products that can be recognized by immune and non-immune cells as Pathogen-associated molecular patterns (PAMPs) and can also cause the release of Damage-associated molecular pattern molecules (DAMPs) (97). PAMPs are structural motifs which serve as “danger” signals to the host indicating the presence of virus that trigger host defenses. These danger signals can be structural proteins and glycolipids but are mainly nucleic acids including double-stranded RNA (dsRNA), viral single-stranded RNA, and CpG DNA (98, 99). DAMPs are host nuclear or cytosolic proteins with defined intracellular function that activate effector cells from the innate immune system when they are released outside the cell (100). Virus-induced changes such as an increase in pro-inflammatory cytokines and chemokines, a decrease in immunosuppressive cytokines, and the release of PAMPs and DAMPs at the site of the tumor may diminish or reverse the established immunosuppressive microenvironment and initiate antitumor immunity.

Several oncolytic virus classes are currently in late-stage clinical trials (Table 3). The most advance of these, Talimogene laherparepvec (T-VEC, formerly OncoVex or JS1/ICP34.5-/ICP47-/GM-CSF; an HSV isolate selected for its potency over laboratory strains, it is deleted in both the ICP34.5 and ICP47 genes to further increase viral replication and tumor cell killing, it also expresses human GM-CSF for immune stimulation) has demonstrated some very promising clinical data. From recently announced results of a phase III trial in unresectable stage IIIB-IV melanoma receiving either T-VEC injected into the lesion or GM-CSF administered subcutaneously, the overall durable response rate (DRR) was 16.3% for T-VEC treated patients as compared to 2.1% for GM-CSF treated individuals (101). The objective overall response rate (ORR) was 26.4% for the T-VEC group (including 10.8% complete responders) compared to an ORR of 5.7% and a complete response rate of 0.7% in the GM-CSF alone group (101). Importantly, in a phase II trial, tumor shrinkage was noted in non-injected lesions, demonstrating that systemic immunity was induced (102). In addition, and across a number of viruses, studies have shown that both innate and adaptive immune responses are generated following viral tumor lysis (92, 103–111). This antitumor immunity is an important outcome of oncolytic viral therapy as it would lead to the destruction of tumor cells that escaped the initial viral lysis.

**Table 3:** **The most advanced clinically evaluated oncolytic viruses**.

Virus	Name	Cancer type	Reference
Adenovirus	ONYX-015 H101	SCCHN	(112–114)
		Glioma	
		Ovarian	
	CGTG-102	Solid tumors	(115)
	CG0070	Bladder	(116, 117)
	ICOVIR-5	Solid tumors	(118–120)
	ColoAd1	Colorectal	(121)
Vaccinia virus	GL-ONC1	Solid tumors	(122–124)
	JX-594	Liver tumors	(125, 126)
		Solid tumors IV	
Herpesvirus	G207	Glioma	(127–129)
	NV1020	Liver tumors IA	(130, 131)
	T-Vec	Breast	(132, 133)
		SCCHN	
		Melanoma IT	
		Liver tumors	
Reovirus	Reolysin	SCCHN IT	(134–136)
		Solid tumors IV	
Measles virus	MV-CEA	Ovarian IP	(137, 138)
	MV-NIS	Ovarian IP	(139–141)
		Glioma IT	
		Myeloma IV	
		Mesothelioma	
NDV	PV701	Solid tumors	(142, 143)

*Above trial information from ClinicalTrials.gov*.

## Merging Oncolytic Viruses and Immune-Checkpoint Blocking

The realization that oncolytic viral therapy can itself be an immunotherapy has in many ways reinvigorated the field and expanded the possible approaches that can be taken to treat cancer. Similarly, the discovery and targeting of immune checkpoints has opened a new immuotherapeutic avenue generating very promising clinical results. The potential to combine oncolytic viruses with a blockade of immune checkpoints is a very exciting strategy that may be beneficial on many levels and help overcome current shortcomings associated with either approach alone. To date, there have been only a few preclinical studies combining oncolytic viruses and immune-checkpoint blockers (anti-CTLA-4 mAb) (144, 145). However, results have been promising with one study showing that replication competent VSV in combination with anti-CTLA-4 mAb resulted in the elimination of macroscopic tumor implants in the majority of test animals, an outcome that could not be achieved by either treatment alone (145). The study went on to show that the response was CD4 and CD8 T-cell mediated (145). When combining these two approaches, the exact virus/checkpoint combination will likely need to be determined empirically with many factors including indication and immune status of patient playing a role. However, in general an argument can be made that the greatest synergies between these strategies would be realized by delivering the immune-checkpoint therapy directly from the oncolytic virus (Table 4).

**Table 4:** **The benefits of using an oncolytic virus to deliver immune-checkpoint blockers**.

Viral attribute	Benefit		
	Safety	Potency	Economic
Immuno-stimulatory		x	
Targeted delivery	x	x	x
Delivery of alternative Ab formats	x	x	x
Multi-gene delivery	x	x	x

### Increased priming and greater immune potency

Preclinical studies have shown that in mice bearing partially immunogenic tumors, treatment with CTLA-4 antibodies could elicit significant antitumor responses whereas poorly immunogenic tumors were refractory to anti-CTLA-4 administration (32, 146). However, these refractory tumors could be made more responsive by administering granulocyte-macrophage colony-stimulating factor (GM-CSF) in combination with the anti-CTLA-4 (146). These findings suggested that a CTLA-4 blockade enhances an already existing endogenous antitumor response resulting in tumor regression. But when the tumor is poorly immunogenic and does not induce a robust enough immune response the anti-immune checkpoint is not as efficacious. Similar results have been found in the clinic where analysis of pre-treatment tumors indicated that patients with high baseline expression levels of immune-related genes were more likely to respond favorably to ipilimumab (147). Just as the GM-CSF is used to help boost the initial innate immune response, oncolytic viruses could have a similar effect as it is clear that the oncolytic viral infection has pro-inflammatory properties, eliciting both an innate and adaptive immune response.

### Enhanced safety and efficacy by expressing immune-checkpoint blockers from the oncolytic virus

The oncolytic virus and the immune-checkpoint blocker could be administered as two separate therapeutics but one of the most appealing aspects of the oncolytic viral approach is that it is localized to the tumor. This localization confers several advantages for both safety and potency. Clinical and preclinical data strongly suggest that a blockade of immune checkpoints is a very potent antitumor therapy. However, there are, in some cases, unwanted side effects. Given the importance of the immune checkpoints in maintaining immune homeostasis there is concern that a blockade of these receptors and/or ligands could lead to a break in immune self-tolerance resulting in autoimmune/autoinflammatory side effects (148). Blocking CTLA-4 as a therapy was initially questioned given its crucial role in the regulation of T-cell amplification. The phenotype of *Ctla4*-knockout mice also hinted at the possibility of a high number of unwanted immune-related effects. In the pivotal phase III trial of ipilimumab, Grade 3 or Grade 4 immune-related adverse events (including rash, colitis, hepatitis, and endocrinopathies) occurred in 10–15% of patients treated with the anti-CTLA-4 antibody as compared to 3% of those treated with gp100 alone. During this trial, there were 14 deaths related to ipilimumab (2.1%), 7 of which were due to immune-related adverse events (17). Delivering the immune-checkpoint blocker (Ab, Ab derivative or modified ligand or receptor) from the oncolytic virus would localize the treatment and mitigate the risks inherent in systemic delivery. In preclinical studies of a replication competent adenovirus armed with the coding region of a full length CTLA-4 antibody a 43-fold higher antibody concentration in the tumor as compared to the plasma was noted (144). Moreover, plasma levels in treated mice remained below the reported human safety threshold (144).

It is also possible to make expression of these immune-checkpoint blockers contingent upon a productive viral infection (i.e., selective replication that is restricted to the tumor cell) further increasing the safety of the therapeutic. This can be done by utilizing endogenous late viral promoters that are dependent upon the uptake and replication within the target tumor cell to express exogenous genes and has been described for human adenovirus (12, 13, 149). In the normal cell, this expression would be blocked as replication would not be achieved consequently confining expression to target cancer cells. Potency, like safety also benefits from this localized delivery, concentrating the therapeutic to the tumor and its microenvironment. Accumulation of virally delivered transgenes (including reporter genes, prodrug converting enzyme, anti-angiogenic factors, immunostimulatory factors) at the site of the infected tumor has been shown in numerous studies (97, 115, 132, 150–153). For example, PET imaging experiments have dramatically demonstrated the tumor localized expression of thymide kinase following infection with an oncolytic virus armed with the enzyme (154, 155). This accumulation was translated into efficacy upon administration of the prodrug Ganciclovir (154). Additionally, the self-perpetuating nature of an oncolytic infection results in sustained transgene expression (156) that will continue until tumor regression is complete and the virus is eliminated from the tumor site by the immune system (157). Therefore the amount of material produced would be directly related, in theory, to the tumor load, personalizing the respective dose to the individual and their tumor burden. It is also appealing to consider that this may eliminate peaks and valleys associated with the intravenous administration of the therapeutic as the virus expressed molecule would be generated on a more constant basis that might also benefit the patient.

### Enablement of alternative therapeutics

Although viruses can be used to deliver an intact IgG, their focused delivery to the tumor site and their self-perpetuating nature allow for the use of alternative antibody formats such as diabodies, Fabs, and scFvs (144, 158). This could have a profound impact on any mAb-based antitumor therapeutic particulary immune-checkpoint blockers. From a safety standpoint, the use of these alternative Ab formats could be beneficial because IgGs, due to their size (150 kDa), have prolonged serum half-lives (>10 days) and are therefore more likely to have associated toxicities. If these alternative formats were to escape the tumor site their faster clearance reduces the risk for off-target events. For immune-checkpoint blockers, this could help to decrease the immune-related adverse events that have been associated with this therapeutic approach (148, 159). Additionally, smaller formats would potentially penetrate the tumor to a greater extent than a full length antibody. Studies have shown that an intact IgG molecule takes 54 h to move 1 mm into a solid tumor, whereas a Fab fragment travels the same distance in only 16 h (160). This enhanced penetration could increase overall efficacy. The diabodies in particular have been shown to provide rapid tissue penetration, high target retention, and rapid blood clearance presumably as a result of their multi-valent nature and intermediate size (55 kDa) (161). The use of alternative antibody formats also opens up the possibility of delivering multiple therapies from one oncolytic virus. This may have broad implications for the blockade of immune-checkpoint approach as studies are beginning to show that targeting multiple checkpoints may be more efficacious (67–71, 74). Without localized delivery, the use of these alternative formats would likely not be feasible as they would clear too rapidly (on the order of a few hours or minutes dependent upon the format) (162). This may necessitate the need for higher input doses or multiple injections of the Ab, which could potentially be cost prohibitive. Having localized delivery via the virus would avoid the need for full length Abs and make the smaller, faster-clearing formats viable therapies that are still capable of efficacious outcomes.

### Economically advantageous

Expression of immune-checkpoint blockers from an Oncolytic virus is economically appealing. If one assumes that the initial promising results seen with combination checkpoint blockers are maintained in larger phase II and III trials, the delivery of a combination of blockers from a virus would eliminate the need to commercially manufacture the molecules separately. This approach utilizes a single entity (the virus) to exploit the natural machinery of the virus and the tumor cell to continuously produce the therapeutic agents so long as the tumor cells continue to exist. Moreover, it has been demonstrated that multiple exogenous proteins can be delivered from a single virus (149). Due to their tumor selective localization, as mentioned previously, they would not need to express a full length antibody, making this approach potentially attractive and novel for delivering multiple-checkpoint inhibitors to the site. In addition, this therapy would have the potential added benefit of increased immunogenicity and/or direct tumor cell lysis offered by the oncolytic virus. Thus expressing a single biological agent with the ability to deliver multiple-checkpoint inhibitors that itself has anti-cancer activity is an interesting possibility. However, it should be kept in mind that the commercial manufacture of oncolytic viruses is behind that of antibodies and thus may be only a true economic advantage in the future with additional optimization.

## Conclusion

In the fight against cancer, no single magic bullet has emerged. Despite several improvements in diagnostics and therapies nearly 7 million cancer-related deaths still occur every year worldwide (163). One reason is that cancer is complex and can evolve to thrive under harsh conditions and to evade the body’s natural defenses. Two promising therapeutic strategies have emerged; the blockade of immune checkpoints and oncolytic viruses and we believe that an argument can be made that the greatest potential for both of these therapies lies in the synergies that would be realized by delivering the immune-checkpoint therapy directly from the oncolytic virus (Table 5). We look forward to the continued evolution of these agents and to the exciting years ahead as we begin to see these agents come forward pre-clinically and clinically.

**Table 5:** **The pros and cons of oncolytic viral, immune-checkpoint inhibition and combination therapy**.

Therapeutic approach	Pros	Cons
Oncolytic virus	Selective for cancer cells	Selectivity is potentially cancer-type dependent
	Self-amplifying therapy	Suboptimal potency as a monotherapy
	Tumor burden dependent	Pro-inflammatory/immunogenic
	Pro-inflammatory/immunogenic	Manufacturing challenges
	Endogenous gene delivery	
Immune-checkpoint inhibitor	Potential to be non-cancer-type specific	Potential for adverse immunological events
	Potent/lasting tumor immunity	Dependent on immune status of patient
	Amendable to current biologics (antibodies, recombinant ligands, receptors)	
Oncolytic virus + immune-checkpoint inhibitor	Selective for cancer cells	Selectivity is potentially cancer-type dependent
	Self-amplifying therapy	Manufacturing challenges
	Tumor burden dependent	
	Pro-inflammatory/immunogenic	
	Endogenous gene delivery	
	Potent/lasting tumor immunity	

## Conflict of Interest Statement

Dr. Terry Hermiston serves on the following boards: Scientific Advisory Board for Psioxus Therapeutics and Board of Directors for Jennerex Biotherapeutics. The other co-author declares that the research was conducted in the absence of any commercial or financial relationships that could be construed as a potential conflict of interest.
